# L-Glutamine Supplementation Alleviates Constipation during Late Gestation of Mini Sows by Modifying the Microbiota Composition in Feces

**DOI:** 10.1155/2017/4862861

**Published:** 2017-03-12

**Authors:** Yuanyuan Zhang, Taofeng Lu, Lingxia Han, Lili Zhao, Yinjie Niu, Hongyan Chen

**Affiliations:** Laboratory Animal and Comparative Medicine, State Key Laboratory of Veterinary Biotechnology, Harbin Veterinary Research Institute, Chinese Academy of Agricultural Sciences, Harbin 150069, China

## Abstract

Constipation occurs frequently in both sows and humans, particularly, during late gestation. The microbial community of the porcine gut, the enteric microbiota, plays a critical role in functions that sustain intestinal health. Hence, microbial regulation during pregnancy may be important to prevent host constipation. The present study was conducted to determine whether L-glutamine (Gln) supplementation improved intestinal function and alleviated constipation by regulation of enteric microbiota. 16S rRNA sequences obtained from fecal samples from 9 constipated sows (3 in the constipation group and 6 in the 1.0% Gln group) were assessed from gestational day 70 to 84. Comparative analysis showed that the abundance of intestinal-friendly microbiota, that is, Bacteroidetes (*P* = 0.007) and Actinobacteria (*P* = 0.037), was comparatively increased in the 1.0% Gln group, while the abundance of pernicious bacteria,* Oscillospira* (*P* < 0.001) and* Treponema *(*P* = 0.011), was decreased. Dietary supplementation with 1.0% Gln may ameliorate constipation of sows by regulated endogenous gut microbiota.

## 1. Introduction

The gut microbiota is a complex and mostly anaerobic ecosystem that plays a key role in the maintenance of health, physiological function, and regulation of disease pathogenesis of the host [1, 2]. There is increasing evidence that microbes in the gastrointestinal tract of the host play an important role in protein and amino acid metabolism [3], as the gut microbiota is reported to alter the metabolic states of weaned piglets [4], broiler chickens [5], children [6], and pregnant women [7]. Constipation occurs in about one-fourth (range, 9%–39%) of women during pregnancy and at 3 months postpartum [7]. Nonetheless, drug use should be avoided during gestation for any reason and not used as a last resort. Thus, alteration to the gut microbiota has been suggested a possible solution to constipation [8], as diet and nutritional status influence the composition and function of gut microbiota and are, therefore, considered potential therapeutic methods to alleviate constipation.

The diet can have a marked impact on the intake of the three main macronutrients (carbohydrates, proteins, and fats) and can significantly affect the composition of the gut microbiota [9]. A combination of different types of fibers into a single product for fiber supplementation may result in greater effectiveness against constipation [10] and soften stool texture. It has been reported that supplementation with functional amino acids (e.g., arginine [11], cysteine [12], L-glutamine (Gln) [13], and leucine [14]) can change the composition of the intestinal microbiota of animals to improve gut health and function. To our knowledge, there was no direct evidence that functional amino acids associated with constipation by regulated gut microbiota.

Gln has attracted much attention as an amino acid nutrient and as a primary metabolic fuel factor for intestinal cells [15, 16], as well as considerable interest as a gut-targeted nutrient, not merely due to its proposed key role in the maintenance of intestinal structure and function, but also regulation of bacterial metabolism in the digestive tract [17]. Gln is a key regulator of bacterial survival and growth in the intestine through modulation of bacterial metabolism of nitrogenous compounds [18, 19]. Also, Gln may affect amino acid utilization and metabolism of bacteria in the small intestine [18], indicating that the small intestine is an important site for metabolism of proteins synthesized by intestinal microbiota [20]. Recent studies have confirmed the presence of microbial amino acid metabolites in urine and feces [3]. From the small intestine to the large intestine, the number of microorganisms is expanded geometrically, indicating that more amino acids are used for the growth of microorganisms, which may be one reason why dietary supplementation with Gln promotes growth and improves feed utilization in animals [19].

Gln is classified as a conditionally essential amino acid because it is utilized at a greater rate than it is synthesized during pregnancy [21]. More importantly than enhanced reproductive performance, Gln supplementation can ameliorate constipation and improve intestinal function by regulation of microflora. The aims of this study were (a) to identify the microflora species in the gut of sows during late pregnancy, (b) to determine the effect of Gln regulation on intestinal microflora, and (c) to evaluate the ability of Gln supplementation to ameliorate constipation.

## 2. Materials and Methods

### 2.1. Animal Experiments

To achieve the objectives of this experiment, nine constipated mini sows in the first farrow at an average of 70 days in pregnancy (DIP) were randomly allotted to individual pens. From 70 to 84 DIP, feed for each sow was restricted to 1.0 kg/day. During this period, the diets of six of the nine mini sows were supplemented with 10 g of Gln/day. Gln was purchased from Amresco LLC (Solon, OH, USA). All diets were formulated to provide similar protein and energy levels to meet or exceed the National Research Council (1998) nutritional requirements for swine. Feeds were dried, ground (0.5 mm), and analyzed for crude nutrients using the Weende analysis described by Naumann and Bassler (1997) [22]. Standard methods were used to analyze the macro and trace elements in the diets (atomic absorption spectrometry: calcium; photometry: phosphorus).

Each pen was equipped with a feeder and nipple to allow the mini sows free access to feed and water. The mechanically ventilated room was maintained at a constant temperature of 22°C–26°C under a 16 h light : 8 h dark cycle.

This study was performed in strict accordance with the recommendations of the Guidelines for the Care and Use of Laboratory Animals of the National Institutes of Health. The study protocol was approved by and carried out in full compliance with the animal welfare guidelines of the Animal Care and Use Committee of the Chinese Academy of Sciences (Registration no. 011063506) [23].

### 2.2. Constipation Scores

A constipation score was determined in nine gestating sows. The degree of constipation was scored according to Oliviero et al. (2010) [24] as very severe (0), severe constipation (1), moderate constipation (2), normal feces (3), fairly soft feces (4), or very soft feces (5). A constipated sow was considered as any sow with a constipation score ranging from very severe constipation (0) to moderate constipation (2); and a nonconstipated sow was considered as any sow with a constipation score of normal feces (3).

### 2.3. Gut Microbiota

Feces from mini sows in the constipation group and 1.0% Gln group were collected on 84 DIP and stored at −80°C until assayed. Three samples from each mini sow were combined for gut microbiota analysis.

Microbial genomic DNA was extracted from each fecal sample (0.2 g) using the cetyltrimethylammonium bromide/sodium dodecyl sulfate method. DNA concentration and purity were evaluated on 1% agarose gels. The extracted DNA was diluted to a concentration of 1 ng/*μ*L using sterile water. After quantification, qualification, mixing, and purification of the PCR products, the V4 region of the 16S rRNA was sequenced using an Illumina MiSeq platform (Novogene, Beijing, China) in accordance with the standard protocol of the manufacturer. Raw data were assembled using the FLASH analysis tool (http://ccb.jhu.edu/software/FLASH/) [25] and filtered with the Quantitative Insights into Microbial Ecology (QIIME) software package (http://qiime.org/) [26]. Chimera sequences were removed with the UCHIME algorithm (http://drive5.com/usearch/manual/uchime_algo.html) [27] to obtain effective tags. Sequences with ≥97% similarity, as analyzed with Uparse software (http://drive5.com/uparse/), were assigned to the same operational taxonomic unit (OTU) [28]. Meanwhile, the Ribosomal Database Project Classifier (http://rdp.cme.msu.edu/) [29] was used to assign each OTU to a taxonomic level. Multiple Sequence Comparison by Log-Expectation (MUSCLE) software (http://www.drive5.com/muscle/) [30] was used to perform multiple sequence alignments. In order to compute alpha diversity to analyze the complexity of species diversity for a sample, observed species were applied. Beta diversity of both the weighted and unweighted UniFrac metrics, which consider the presence/absence of taxa between sample pairs, was calculated using QIIME software. Cluster analysis was preceded by principal component analysis. Principal coordinate analysis (PCoA) was performed to visualize principal coordinates from complex, multidimensional data. The unweighted pair-group method with arithmetic means (UPGMA) hierarchical clustering was to interpret the distance matrix using average linkages. All original sequences were downloaded from the National Center for Biotechnology Information database (https://www.ncbi.nlm.nih.gov/pubmed/).

### 2.4. Statistical Analysis

Statistical analyses were performed using the SPSS software 19.0 (IBM-SPSS, Inc., Chicago, IL, USA). Frequency analyses were conducted for constipation score data. The constipation score was compared in the middle of and after treatment using Wilcoxon's rank sum test. The data were presented as median and range of the variables. The percentages of sows with constipation (scored 1 to 3) were compared among days and groups by using chi-square test. *P* < 0.05 was regarded to be statistically significant.

## 3. Results

### 3.1. Feed Composition

The analyzed compositions of the diets are shown in Table 1. There were no significant differences (*P* > 0.05) in the feed composition between constipation and 1.0% Gln groups.

### 3.2. Constipation Scores

Determination of constipation score in nine gestating sows varied as severe constipation (1), moderate constipation (2), and normal feces (3). At the beginning of the experiment, nine gestating sows of two groups were all scored 1. Constipation scores in the middle and at the end of the experiment were displayed in Table 2. Three sows from constipation group had scored 1 during the whole experiment. In treatment group, supplied with 1.0% Gln each day, one sow had scored 3, and four sows had scored 2 in the middle of experiment. At the end of experiment, 50% of the sows from 1.0% Gln group had a constipation problem scored 2, while the rest were recovery scored 3. The constipation score increased significantly in the middle and at the end of the experiment (*P* < 0.05).

### 3.3. Sequencing Data

Sequencing information and estimators of richness were summarized in Table 3. The Illumina-based analysis of the V4 region of 16S rRNA gene produced 493. 362 total tags for bacteria. After filtering and removing potential erroneous sequences, a total of 478,044 effective tags were obtained for bacteria. Based on 97% similarity, an average of 735 and 706 OTUs (operational taxonomic units) for bacterial diversity was obtained in constipation group and 1.0% Gln group, respectively.

**Figure 1:**
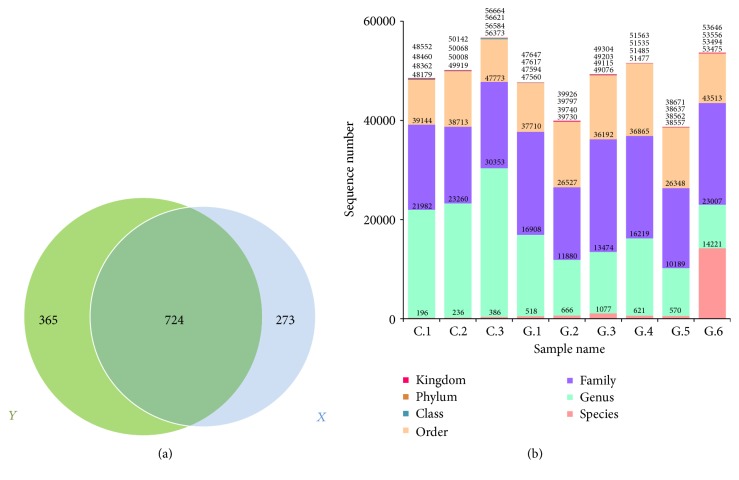
(a) A Venn diagram of OTUs. The letter “*X*” represents the number of OTUs in constipated pregnant mini sows and “*Y*” represents the number of OTUs in constipated pregnant mini sows fed a diet supplemented with 1.0% Gln. Unique and shared OTUs between the two groups were based on 97% similarity. (b) The number of taxon tags at different levels.

A Venn diagram of the OTUs is presented in Figure 1(a). Because the main nutrient composition of diet was similar, there were about 724 similar OTUs between the constipation and 1.0% Gln groups. The unique amounts of OTUs in the two groups were 273 and 365, respectively. Gln supplementation obviously enhanced the number of OTUs from 997 to 1089. To analyze the type of tags, the amounts of tags classified at levels of kingdom, phylum, class, order, family, genus, and species are presented in Figure 1(b). It can be seen that the quantity of tags at the phylum, order, and genus levels was different between two groups.

**Figure 2:**
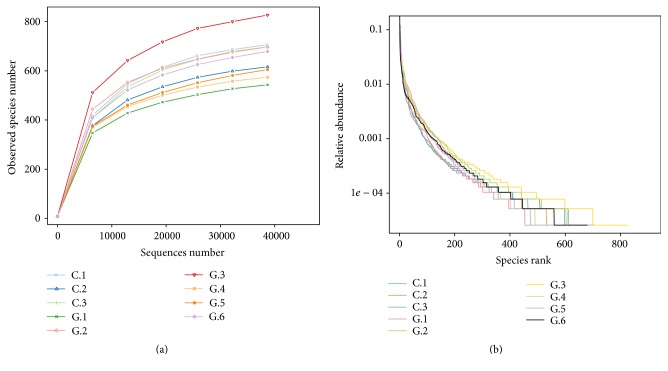
(a) Species rarefaction curves of all samples. (b) Rank-abundance curves of all samples.

Species rarefaction curves are shown in Figure 2(a). With the sequencing number constantly growing, the curves of the observed species were initially very steep and then gradually increased. All curves tended to be flat at the end, which illustrated that the existing sequencing data volume was reasonable to detect a sufficient number of species. 16S rRNA-based high throughput sequencing was used to reveal the composition and abundance of microbial communities. As shown in Figure 2(b), the rank-abundance curve showed similar richness and evenness of microbial species in all nine samples. Due to the complex nutrient content in feces, the slopes of all curves were small, which indicated a high degree of evenness among the microbial species.

### 3.4. Microbial Community Analysis

Bacterial tags of constipation and 0.1% Gln group covered more than 32 phyla, 81 classes, 118 orders, 168 families, and 195 genera. Bacterial sequences from the two groups were further analyzed at the phylum, order, and genus levels. Phyla with a relative abundance of ≥0.1% were considered predominant. Sequences that failed to be classified or phyla with a relative abundance of <0.1% were assigned as “Other.”

**Figure 3:**
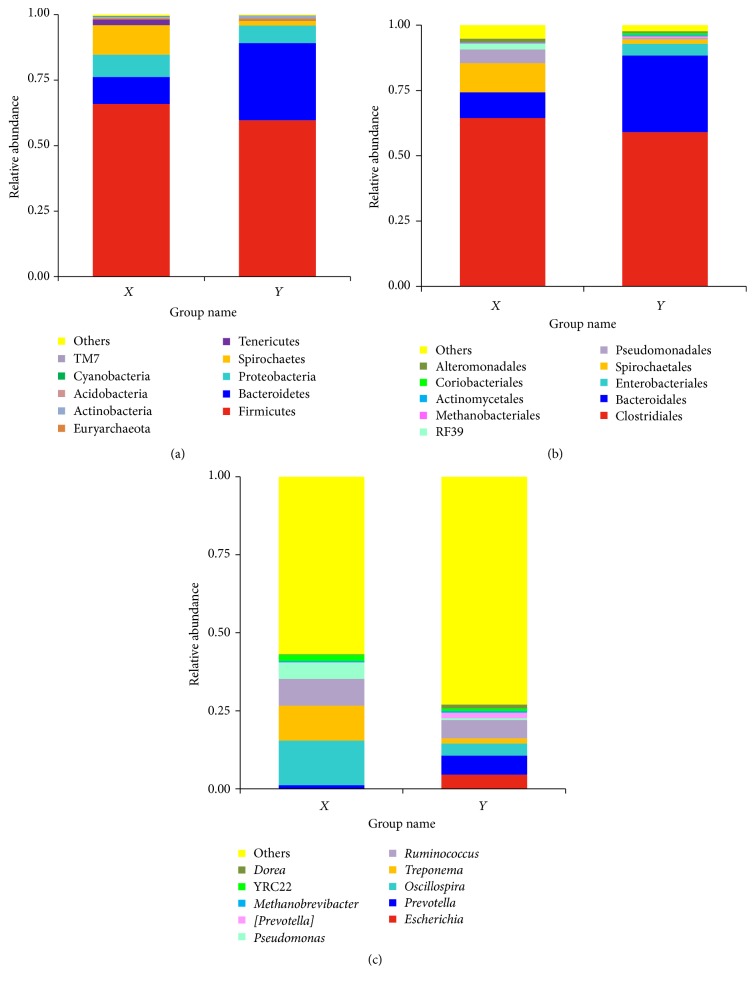
Relative abundance of *X* (represents constipation group) and *Y* (represents 1.0% Gln group) at the phylum level (a), order level (b), and genus level (c). Each color represents the percentage of the levels in the total effective tags of each group.

Based on average abundance analysis, at the phylum level, Firmicutes (65.90%), Bacteroidetes (10.31%), Proteobacteria (8.49%), Spirochaetes (11.25%), and Tenericutes (2.20%) were the five major phyla of bacteria in constipation group (Figure 3(a)). Firmicutes (59.67%), Bacteroidetes (29.46%), Proteobacteria (6.67%), and Spirochaetes (1.99%) were the four major phyla of bacteria in 0.1% Gln group (Figure 3(a)). In addition, Euryarchaeota, Actinobacteria, Acidobacteria, Cyanobacteria, TM7, and other bacteria were found in two groups with low abundance (<1%) (Figure 3(a)). Group of 0.1% Gln had higher abundance of Bacteroidetes (*P* = 0.007) and Actinobacteria (*P* = 0.037) and lower abundance of Spirochaetes (*P* = 0.012) than constipation group.

Based on average abundance analysis, at the order level, Clostridiales (64.44% for constipation group and 59.10% for 0.1% Gln group), Bacteroidales (9.79% for constipation group and 29.29% for 0.1% Gln group), and Spirochaetales (11.18% for constipation group and 1.67% for 0.1% Gln group) were the three major orders of bacteria in the two groups (Figure 3(b)). In addition, Enterobacteriales, Spirochaetales, Pseudomonadales, RF39, Methanobacteriales, Actinomycetales, Coriobacteriales, Alteromonadales, and others were found with low abundance in the two groups (Figure 3(b)). Group of 0.1% Gln had higher abundance of Bacteroidetes (*P* = 0.007) and lower abundance of Spirochaetes (*P* = 0.011) than constipation group.

Based on average abundance analysis, at the genus level,* Prevotella* (1.11% and 6.12%),* Oscillospira* (14.32% and 3.90%),* Treponema* (11.18% and 1.67%), and* Ruminococcus* (8.60% and 5.82%) were the four major genera of bacteria in constipation group and 0.1% Gln group, respectively (Figure 3(c)). In addition,* Escherichia*,* Pseudomonas*,* Prevotella*,* Methanobrevibacter*,* YRC22*,* Dorea*, and other bacteria were found in two groups with low abundance (<1%) (Figure 3(c)). Group of 0.1% Gln had higher abundance of* Dorea* (*P* < 0.001) and lower abundance of* Oscillospira* (*P* < 0.001) and* Treponema* (*P* = 0.011) than constipation group.

### 3.5. Beta Diversity Index Analysis

**Figure 4:**
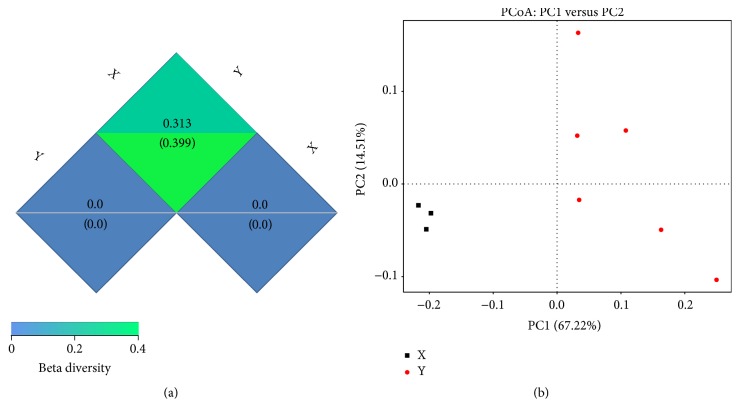
(a) Beta diversity (weighted UniFrac) among two groups. The letter “*X*” represents diversity of the constipation group and “*Y*” represents diversity of the 1.0% Gln group. Beta diversity indexes were measured based on weighted UniFrac and unweighted UniFrac distances. The upper and lower numbers in the grid represent the weighted UniFrac and unweighted UniFrac distances, respectively. (b) PCoA plot based on the weighted UniFrac metric. The letter “*X*” represents clusters of the constipation group and “*Y*” represents clusters of the 1.0% Gln group.

The results of beta diversity (weighted UniFrac) are shown in Figure 4(a). Based on the weighted UniFrac distance cluster analysis, a dissimilarity coefficient for the nine samples was measured to estimate the divergence of microbial species between them. The lower dissimilarity coefficients suggest the less divergence of microbial species. In this study, the dissimilarity coefficients were measured to be 0.313 bacterial diversities, suggesting that divergence between two groups was observed for bacterial species.

PCoA based on the weighted UniFrac algorithm clearly revealed that the feces microbial community varied between two groups (Figure 4(b)). With or without 1.0% Gln addition, it was noteworthy that the samples could be grouped into two distinct clusters. No exceptions from both study groups were observed, reflecting that there was no influence of other genetic and environmental factors on the gut microbiome.

## 4. Discussion

Constipation is a common symptom during pregnancy that affects about half of all women both during pregnancy and up to 3 months postpartum [7]. It has been proposed that an elevation in circulating progesterone slows gastrointestinal motility [31] and fetal growth in late pregnancy, resulting in intestinal malrotation [32]. With mechanical changes in gestation, constipation would be most likely to exert its influence in the third trimester [33]. Both in the laboratory and feedlot, pregnant sows are housed in crates. Low matrix activity and the relative high volume of a fetus generally result in constipation in late pregnancy. Also, constipated sows develop mastitis at higher rates than those without constipation [34]. Our results showed that Gln supplementation group had significantly higher constipation score values than did the sows of the constipation group. The findings of this study lay a technical foundation to further investigate factors associated with constipation during late pregnancy in sows, while providing a reference for human therapies.

Diet is recognized as one of the most influential factors of constipation [9, 35]. Functional amino acids, including Gln, are important regulators of key metabolic pathways that are crucial for maintenance, growth, reproduction, immune function, and intestinal health [21]. As the most abundant and the most accreted amino acid in the second third of gestation, Gln may reduce variation in birth weights [36] and improve placental development and fetal growthunder various physiological and pathophysiological situations in both humans and other mammals [22, 37]. In sows, 60% of fetal growth occurs in the last 24 days of gestation (i.e., days 90–114), which is associated with an accelerated need for Gln by the fetus [35, 38]. Supplementation with 1.0% Gln to the swine diet during late gestation ameliorates fetal growth retardation in gilts and reduces preweaning mortality of piglets [35]. Also, 1.0% Gln supplementation to the diets of postweaned rabbit decreased fattening mortality and modified intestinal microbiota [13]. Gln plays an important role in nitrogen balance and protein synthesis in resident bacteria of the small intestine [19]. These results provide a strategy for Gln supplementation to enhance the productivity and performance of pregnant animals and, more importantly, regulation of intestinal microbiota function.

The microbiota residing in the intestinal tract produces a great variety of compounds from the metabolism of dietary and endogenous substrates that could affect host physiology [1, 39]. The anatomy, genetics, and physiology of pigs are very similar to those of humans. Therefore, a porcine model is often used to study human intestinal digestibility and gut ecology [40, 41]. Fecal samples are easy to obtain and are suitable materials to characterize intestinal microbes in many applications [42]. In the present study, 16S rRNA sequences were used to characterize fecal microbial communities and to identify differences in microbial communities between subjects and groups. As confirmed in a larger study, autologous gut microbes contribute to the development and amelioration of constipation [43].

Members of the phyla Bacteroidetes, Spirochaetes, and Actinobacteria largely accounted for the significant changes observed between the constipation and 1.0% Gln groups. The abundance of Bacteroidetes was significantly decreased in the gut of the constipated patients [44]. Our data showed a trend of increased proportions of Bacteroidetes and Actinobacteria at the phyla level with 1.0% Gln supplementation. Although the abundance of each phylum in mammals fluctuated and was influenced by multiple factors, such as animal species, diet, and pregnancy, Firmicutes and Bacteroidetes were the dominant phyla in this study, followed by Fusobacteria, Proteobacteria, and Actinobacteria [45]. Owing to increase in Bacteroidetes, Gln supplementation decreased the ratio of Firmicutes to Bacteroidetes. It has been reported that there are more Firmicutes, fewer Bacteroidetes, and consequently a higher Firmicutes/Bacteroidetes ratio in the gastrointestinal tract of obese mice and humans [46]. Pourhoseingholi et al. (2009) found that about 60% of patients with functional constipation were overweight [47]. Gln supplementation may ameliorate constipation by increasing the energy harvesting capacity by Firmicutes species [48]. Additionally, obese pigs had a higher abundance of Spirochaetes than lean pigs [49], and* Bifidobacterium* and* Bacteroides* species were significantly less abundant in feces from patients with functional constipation, as compared with healthy controls [8], which is in agreement with results of the present study. Human adult and infant distal gut microbiomes had significantly higher abundance of Actinobacteria than did the swine microbiome [50]. Zhu et al. (2014) [44] did not detect phyla Acidobacteria in the human gut microbiome of the constipated obese children, which was contrary to our results. This may be due to different species and age.

Corresponding to phylum Bacteroidetes, member of the order Bacteroidales was significantly increased in the gut of the 0.1% Gln group. The decreased abundance of Bacteroidales was associated with obesity [51] and pediatric Crohn's disease [52]. Thus, it was suggested that the increase of Bacteroidales in 0.1% Gln group played the important role in alleviating constipation. The swine fecal metagenome harbored significantly more Spirochetes than the fish and the termite [50]. The decrease of Spirochaetales was beneficial for relieving constipation symptom as our results displayed.

*Treponema* species are members of the family Spirochaetaceae within the order Spirochaetales. Gln has a potentially beneficial effect by protecting pigs against the opportunistic pathogen* Treponema*. Gophna et al. (2017) [53] found that the raw potato starch significantly decreased the relative abundance of* Treponema* and* Oscillospira* in colonic digesta and mucosa of pigs, which suggests that the resistant starch can affect the pigs through the interaction between mucosa-associated microbiota and the host cell, not just its fermentation products. The presence of* Oscillospira* species has an impact on the metabolism of nutritional fibers [54] and has also been shown to be negatively associated with looser stools [55].* Oscillospira* has been linked to gallstones [56], for which slow-transit/constipation is a well-established risk factor.

In summary, the results of this study showed that feeding constipated sows with 1.0% Gln in late pregnancy ameliorated constipation by the regulation of gut microbiota, which has relevance for gut health and function. The results of this study provide evidence that the abundance of endogenous gut microbiota, especially Bacteroidetes and Spirochaetes, may be potentially improved by supplementation with Gln, which can stimulate the growth of beneficial bacteria and suppress replication of potentially harmful microorganisms.

## Figures and Tables

**Table 1:** Analyzed composition of the basal rations for mini sows used in the experiment.

Analyzed composition	Groups^1^	
	*X*	*Y*
Dry matter (DM) (%)	92.33	92.42
Crude protein (%)	13.56	13.59
Crude fat (%)	5.29	5.22
Crude fibre (%)	3.23	3.26
Calcium (%)	0.97	0.98
Phosphorus (%)	0.78	0.78
Available phosphorus (%)	0.44	0.44
Digestible energy (MJ/kg DM)	12.76	12.75

^1^ *X*: constipation group; *Y*: 1.0% Gln group, constipation sows with 10 g of Gln/kg feed·day.

**Table 2:** Effect of 1.0% Gln on gestating sows' constipation scores.

Days of observation	Groups^2^	Number of sows	Constipation score	Percentage of sows with constipation^1^
77	*X*	3	1.00 ± 0.00^b^	100.00^a^
	*Y*	6	2.00 ± 0.26^a^	83.33^b^
84	*X*	3	1.00 ± 0.00^b^	100.00^a^
	*Y*	6	2.50 ± 0.22^a^	50.00^c^

Different superscript letters within a column differ significantly (*P* < 0.05); ^1^Constipation was considered as any sow with a constipation score ranging from severe constipation (1), moderate constipation (2) and non-constipated sow (3), ^2^*X*: constipation group; *Y*: 1.0% Gln group, constipation sows with 10 g of Gln/kg feed·day.

**Table 3:** Sequencing information in this study.

Sequencing information	Groups^1^								
	*X*			*Y*					
	C.1	C.2	C.3	G.1	G.2	G.3	G.4	G.5	G.6
Number of total tags	49991	52233	60048	58092	50646	55283	57652	49447	59970
Number of effective tags	49523	50779	58123	56200	48431	53565	56265	47482	57676
OTUs (97% similarity)	767	663	775	601	754	893	638	605	749

^1^ *X*: constipation group; *Y*: 1.0% Gln group, constipation sows with 10 g of Gln/kg feed·day.
